# The role of IL-18 and IL-33 in the bronchoalveolar lavage fluid of children with severe community-acquired pneumonia complicated with pleural effusion

**DOI:** 10.3389/fped.2025.1560328

**Published:** 2025-04-25

**Authors:** Yinxia Zhang, Zihao Liu, Yiwei Hong, Li Li, Youzhuo Liang, Liangxin Lin, Wenjian Wang, Heping Wang

**Affiliations:** ^1^ Shantou University Medicine College, Shantou University, Shantou, China; ^2^ Department of Clinical Laboratory, Baoan Central Hospital of Shenzhen, Shenzhen, China; ^3^ Department of Respiratory Diseases, Shenzhen Children’s Hospital, Shenzhen, China; ^4^ Department of Rheumatology and Immunology, Shenzhen Futian Hospital for Rheumatic Diseases, Shenzhen, China

**Keywords:** children, severe community-acquired pneumonia, IL-1 family, pleural effusion, *Haemophilus influenzae*

## Abstract

**Background:**

To investigate the evaluative role of interleukin (IL)-1 family cytokines in bronchoalveolar lavage fluid (BALF) among children with severe community-acquired pneumonia (SCAP) and identify cytokines with clinical relevance for pediatric SCAP.

**Methods:**

Children with SCAP hospitalized at Shenzhen Children's Hospital (2019–2020) were studied. IL-1 family cytokines in the BALF were measured via CBA or ELISA. These cytokines included nine IL-1 family members (IL-1*α*, IL-1β, IL-1Ra, IL-33, IL-18, IL-37, IL-36*α*, IL-36Ra, and IL-38) and two receptors (sST2 and IL-18BP). The ratio of proinflammatory cytokines to anti-inflammatory cytokines was analyzed.

**Results:**

In the BALF of children with SCAP complicated with pleural effusion (PE), the levels of IL-18, the IL-18/IL-38 ratio, and the IL-33 level were significantly elevated (*P* < 0.05). Furthermore, the receiver operating characteristic (ROC) curve indicated that these three markers have strong predictive efficacy for diagnosing SCAP complicated with PE. The levels of members of the IL-1 family, including IL-1*α*, IL-1β, IL-1Ra, IL-18, and IL-33, and their associated ratios significantly differed across different pathogen groups (*P* < 0.05). IL-36*α* and the IL-36*α*/IL-38 ratio differed significantly between the *Haemophilus influenzae* (Hi)-positive and -negative groups (*P* < 0.0001 and 0.0048), with lower levels in the Hi-positive group.

**Conclusion:**

IL-18, IL-33, and IL-38 in BALF may serve as effective markers for predicting the development of PE in pediatric SCAP patients. Additionally, respiratory tract colonization by Hi may diminish the production of specific proinflammatory cytokines, including IL-18, IL-33, and IL-36*α*, during SCAP.

## 1 Introduction

Community-acquired pneumonia is an infection of the lung parenchyma acquired in the community that presents with symptoms and signs of lung infection. It is among the most common causes of hospitalization for children in developed countries (1) and is a major contributor to child mortality in developing nations (2, 3). The incidence of severe community-acquired pneumonia (SCAP) is closely associated with factors such as immune imbalance and cytokine secretion dysregulation, with an excessively dysregulated immune inflammatory response playing a key role in the pathogenesis of SCAP (4). Consequently, early identification of biomarkers that can predict complications in children with SCAP and rapidly identify pathogens is vital for the clinical diagnosis and treatment of SCAP.

Research highlights the crucial role of the IL-1 family in innate immune responses and the development of adaptive responses against invading pathogens, which serve as the primary defense against these threats (5). Their release in the bloodstream is regarded as an early marker of sepsis, aiding in the identification of patients at increased risk of adverse outcomes (6). Furthermore, the IL-1 family is closely associated with abnormal inflammation and significant immune-mediated diseases (5), comprising 11 cytokines with analogous structures and receptor-binding patterns (7, 8), and the IL-1 family engages with a total of 10 receptors and coreceptors. Studies have demonstrated that alterations in multiple IL-1 family cytokines are closely associated with the onset and progression of pulmonary and inflammatory diseases (5, 9, 10). Huang et al. (11) reported that in COVID-19 patients, the levels of circulating inflammatory cytokines of the IL-1 family and secondary cytokines such as IL-6 (known as a cytokine storm) are significantly increased. However, research on the relationship between the IL-1 family and SCAP remains rare, with most studies relying on blood samples. In contrast, cytokine levels in BALF can more accurately reflect pulmonary lesions. Bronchoscopy to obtain BALF for pertinent examinations can facilitate SCAP recovery, etiological analysis, and pathogen detection (12). Owing to the strong local response, alterations in cytokines from BALF are more indicative of disease prognosis.

This study aimed to investigate the role of IL-1 family cytokines in the BALF of children with SCAP complicated with PE. Additionally, it seeks to analyze the differences in the expression of IL1 family in the BALF of pediatric SCAPs infected with different pathogens, in order to find out the cytokines with clinical significance for SCAP in children.

## 2 Methods

### 2.1 Study subjects and grouping

**Figure 1:**
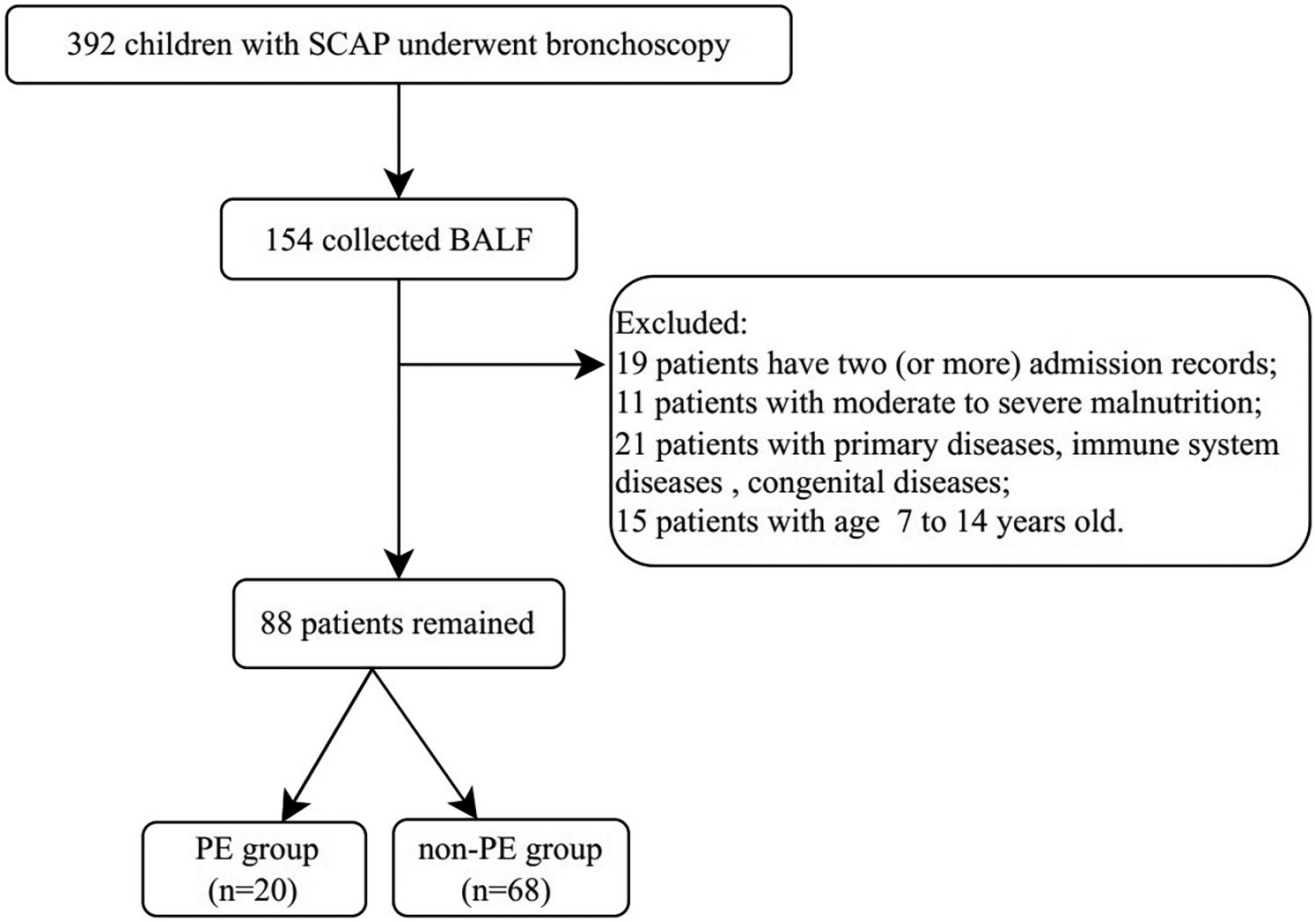
Flowchart of case screening process.

This study involved children hospitalized at Shenzhen Children's Hospital from 2019 to 2020 with a diagnosis of SCAP. The inclusion criterion was in accordance with the standard “Guidelines for the Management of Community-Acquired Pneumonia in Children” (2013 revision) (13), and bronchoscopy had been performed. The exclusion criteria included severe, life-threatening primary diseases of the cerebrovascular, liver, kidney, or hematopoietic system; immune system diseases or malignant tumors; congenital heart and lung defects; moderate to severe malnutrition; and the absence of alveolar lavage fluid collection. The process for case screening is shown in Figure 1.

The study participants were classified into the PE group and the non-PE group on the basis of the presence of PE. The etiological test results were further divided into virus-positive and virus-negative groups, *Mycoplasma pneumoniae* (MP)-positive and -negative groups, bacteria-positive and -negative groups, and *Haemophilus influenzae* (Hi)-positive and -negative groups.

### 2.2 General clinical data collection

General information, clinical manifestations, etiological assessments, radiological data and other data of the subjects were retrospectively collected. Chest x-ray or chest CT combined with chest ultrasound suggested the presence of PE. The etiological and imaging findings were derived exclusively from initial examinations postadmission. Etiological results included 11 pathogen tests from BALF and standard bacterial culture results from sputum.

### 2.3 BALF collection

Following the institutional standard practice (14), the anesthesiologist performed sedation and local anesthesia before bronchoscopy, the respiratory physician used an age-appropriate pediatric flexible fiberoptic bronchoscope to perform lavage of the patient's lungs. Saline (1 ml/kg) was used for bronchoalveolar lavage. The BALF was reclaimed and stored at −80°C for further use.

### 2.4 Cytokine detection methods

The BALF samples from the selected pediatric patients were sourced from the Shenzhen Children's Hospital sample repository and transported to the laboratory for cytokine analysis under optimal low-temperature conditions for cytokine analysis.

The detected cytokines include IL-1*α*, IL-1β, IL-1Ra, IL-18, IL-33, IL-37, IL-36*α*, IL-36Ra, and IL-38, along with interleukin-1 family receptors, including soluble suppression of tumorigenicity (sST2) and interleukin-18 binding protein (IL-18BP). This study employed both cytometric bead array (CBA) and enzyme-linked immunosorbent assay (ELISA) methods for detection.

CBA Method: A LEGEND plex multianalyte detection kit from Biolegend was used to detect cytokines, such as IL-1*α*, IL-1β, IL-18, and IL-33, adhering strictly to the kit's instructions. Detection was performed using a BD FACSCanto II flow cytometer. Post-acquisition, the data were exported, and specialized software was used to determine the concentration of each cytokine on the basis of standard references.

ELISA Method: R&D Systems® ELISA kits were used to detect cytokines, such as IL-1Ra, IL-37, IL-36*α*, IL-36Ra, IL-38, sST2, and IL-18BP, adhering strictly to the kit's instructions. The absorbance (OD value) of each well was measured on an enzyme microplate reader at a wavelength of 450 nm. The standard curve linear regression equation was derived from the standard concentrations and their OD values, enabling the calculation of the concentration for each corresponding indicator.

### 2.5 Statistical analysis

To mitigate the effects of discrepancies in BALF sample recovery rates and to investigate the balance between local proinflammatory and anti-inflammatory cytokines, this study introduces the ratio of proinflammatory to anti-inflammatory cytokines for a more precise reflection of changes in cytokine levels. The specific ratios analyzed included IL-1*α*/IL-38, IL-1β/IL-38, IL-18/IL-38, IL-18/IL-18BP, IL-33/IL-38, IL-33/sST2, and IL-36*α*/IL-38. Given the high concentration of IL-1Ra, which results in minuscule calculated values, the ratio was adjusted to IL-1Ra/IL-1*α* and IL-1Ra/(IL-1*α*+IL-1β) for more straightforward analysis.

Data analysis was performed via SPSS 25.0 statistical software. Categorical variables were presented as counts with percentages, and analyzed using the chi-square test. For normally distributed data, the mean ± standard deviation (SD) was utilized, and comparisons between two groups were conducted via an independent sample *t*-test. For nonnormally distributed data, the median (interquartile range) was employed, and group comparisons were carried out via the Mann‒Whitney *U*-test. The analysis indicated that all cytokines and their ratios in this study exhibited a nonnormal distribution; therefore, all group comparisons were performed via the Mann‒Whitney *U*-test. Additionally, receiver operating characteristic (ROC) curves were generated to assess the predictive and diagnostic value of indicators for SCAP complicated with PE.

## 3 Results

### 3.1 General conditions

#### 3.1.1 General conditions of children with SCAP

Totally, 88 children with SCAP were included. The median time to perform alveolar lavage after admission was 4 (3,6) days. All pediatric patients received medication therapy prior to undergoing bronchoscopy. Baseline characteristics are summarized in Table 1. In this study, 20 patients had complications with PE. On the basis of the presence of PE, the study participants were classified into the PE group and the non-PE group. The clinical Characteristics were presented in Table 2.

**Table 1:** Baseline characteristics of study population.

Characteristics	Number (%)/median (P25, P75)
Gender (male)	61 (69.3)
Age group	
Infancy (<1 year old)	16 (18.2)
Early childhood (<3 years old)	39 (44.3)
Preschool (<7 years old)	33 (37.5)
Duration of hospitalization	8.5 (7,10)^a^
>7 days	56 (63.6)
≤7 days	32 (36.4)
Medication therapy	
Antibiotics	85 (96.6)
Corticosteroid	54 (61.4)
Immunoglobulins	36 (40.9)
Complications	
PE	20 (22.7)
Extrapulmonary complications	14(15.9)

^a^ indicated data that is not normally distributed, expressed as median (P25, P75).

**Table 2:** Comparison of characteristics the PE group and the non-PE group.

Characteristics	PE *n* = 20(%)	non-PE *n* = 68(%)	statistical measure	*P-value*
Gender (male)	13 (65.0)	48 (70.6)	*χ*^2^ = 0.227	0.634
Age group				
Infancy (<1 year old)	4 (20.0)	12 (17.6)	χ^2^ = 10.287	0.006*
Early childhood (<3 years old)	3 (15.0)	36 (53.0)		
Preschool (<7 years old)	13 (65.0)	20 (29.4)		
Duration of hospitalization	9 (8,11.5)	7.5 (7,8)	Z = −1.609^a^	0.108
>7 days	16 (80.0)	40 (58.8)	χ^2^ = 2.995	0.084
≤7 days	4 (20.0)	28 (41.2)		
Medication therapy				
Antibiotics	20 (100.0)	65 (95.6)	χ^2^ = 0.913	1.00
Corticosteroid	14 (70.0)	40 (58.8)	χ^2^ = 0.814	0.367
Immunoglobulins	10 (50.0)	26 (38.2)	χ^2^ = 0.885	0.347

* Represented statistically significant differences between different groups.

^a^ Indicated data that is not normally distributed, expressed as median (P25, P75), and the statistical measure was the *Z*-value.

#### 3.1.2 Etiological analysis of children with SCAP

All pediatric patients underwent 11 pathogen tests for BALF samples and general bacterial culture for sputum samples. The results revealed no pathogens in 7 patients, a single pathogen in 32 patients, and multiple pathogens in 49 patients. A total of 149 pathogens were identified, comprising 70 viruses, 45 MP, 33 bacteria, and 1 fungus. The three most frequently detected pathogens were MP (45 cases), adenovirus (29 cases), and Hi (17 cases).

### 3.2 Comparison of cytokines in the BALF of patients in different groups

#### 3.2.1 Comparison of cytokines in the BALF between the PE group and the non-PE group

**Figure 2:**
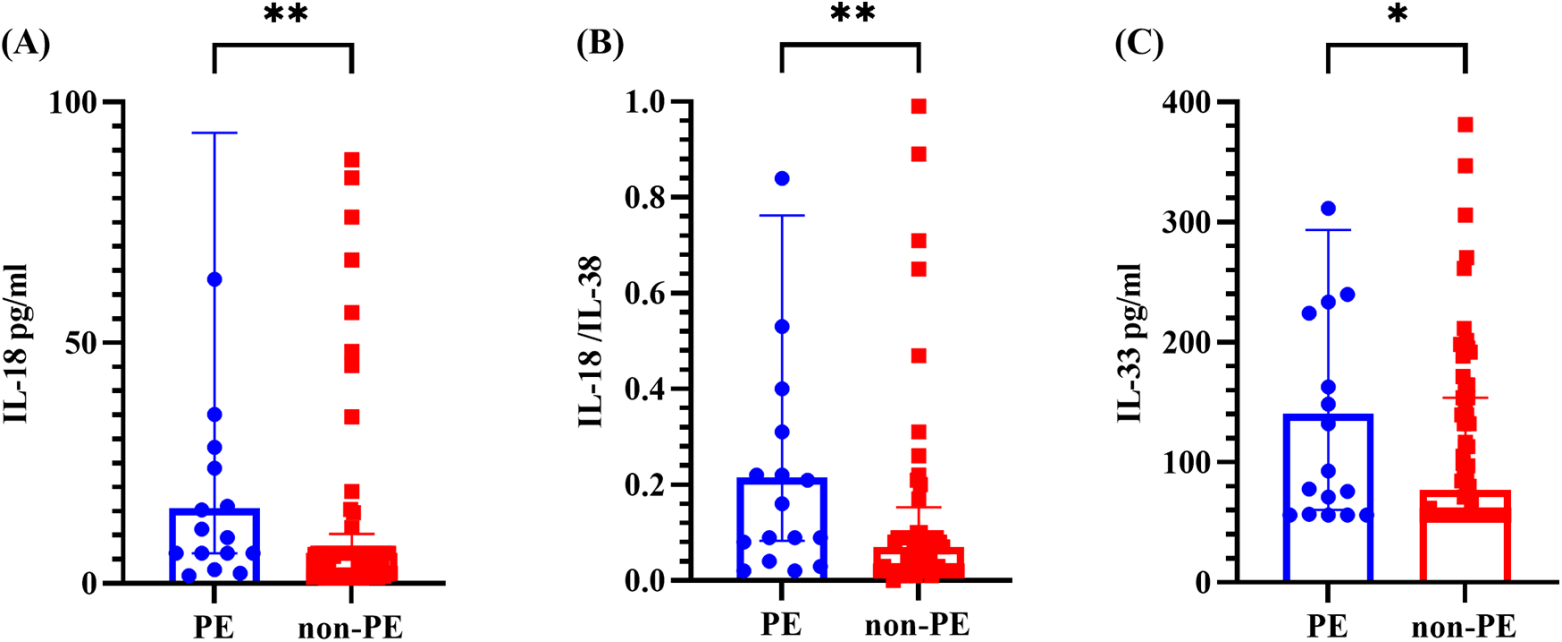
Cytokine levels in the BALF of the PE group and non-PE group. **(A–C)** represents for the levels of IL-18, IL-18/IL-38 ratio and IL-33, respectively. The graph represents the median and interquartile range. * indicated *P* < 0.05, ** indicated *P* < 0.01, comparing the PE group and non-PE group. PE indicated pleural effusion, and non-PE indicated no pleural effusion.

As shown in Figure 2, the levels of IL-18, the IL-18/IL-38 ratio, and IL-33 in the PE group were markedly greater than those in the non-PE group, with all differences reaching statistical significance (*P* < 0.05). No statistically significant differences were observed in the other cytokines or ratios (refer to Supplementary Figure S1).

#### 3.2.2 Comparison of cytokines in BALF between the MP-positive and MP-negative groups

**Figure 3:**
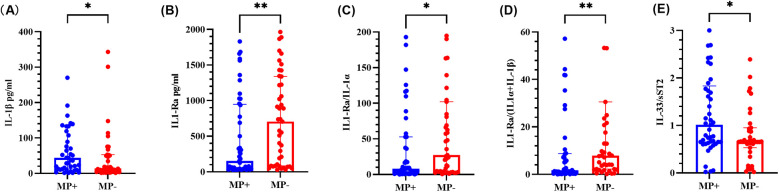
Cytokine levels in BALF from the MP-positive and MP-negative groups. **(A–E)** represents for the levels of IL-1β, IL-1Ra, IL-1Ra/IL-1α ratio, IL-1Ra/ (IL-1α + IL-1β) ratio and IL-33/sST2 ratio, respectively. The graph represents the median and interquartile range. * indicated *P* < 0.05, ** indicated *P* < 0.01. MP + indicated *Mycoplasma pneumoniae* positive, and MP- indicated *Mycoplasma pneumoniae* negative.

As shown in Figure 3, the IL-1β level and IL-33/sST2 ratio were significantly greater than those in the MP-negative group, whereas the IL-1Ra level, the IL-1Ra/IL-1*α* ratio, and the IL-1Ra/(IL-1*α* + IL-1β) ratio were significantly lower (*P* < 0.05). Other cytokines and ratios did not significantly differ (see Supplementary Figure S2).

#### 3.2.3 Comparison of cytokines in BALF between positive and negative groups with different pathogen types

**Figure 4:**
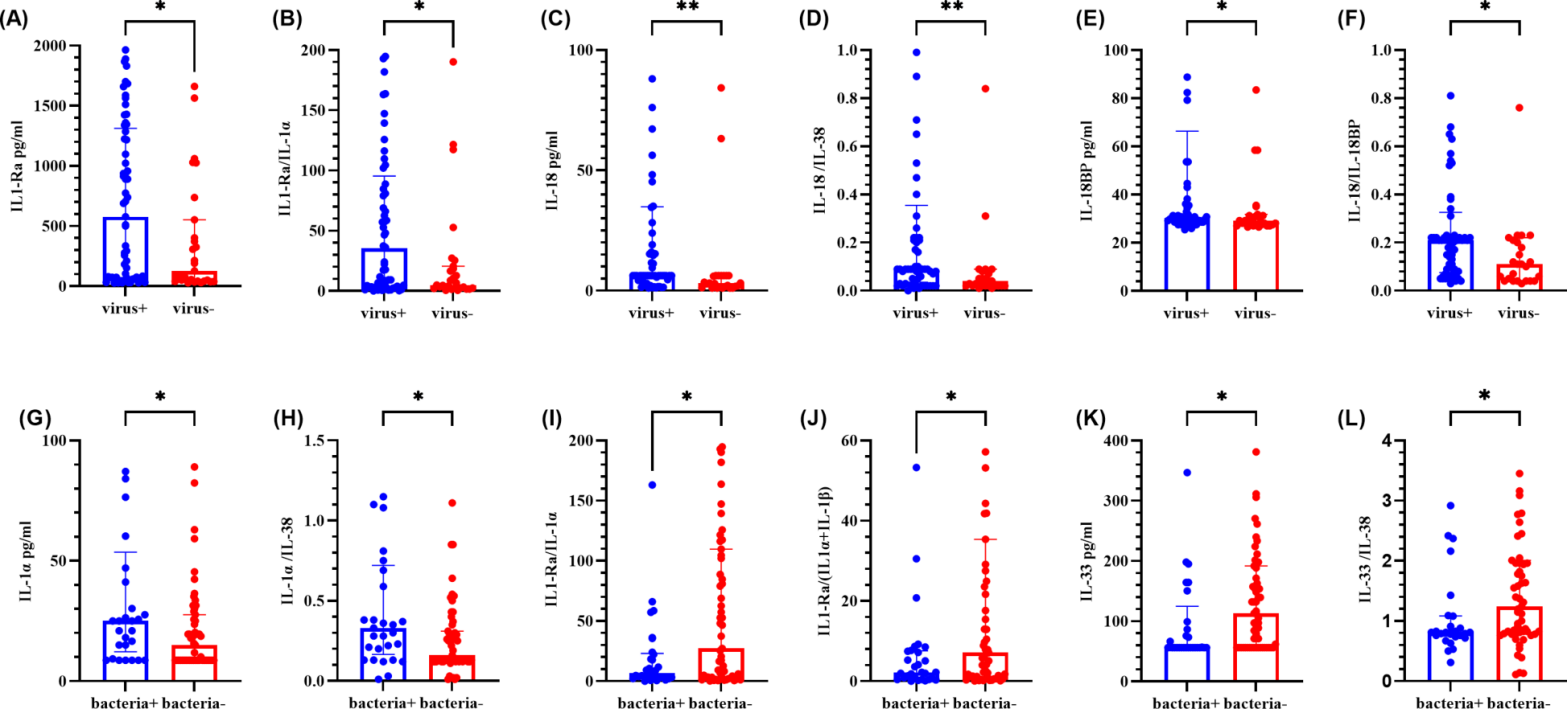
Comparison of cytokine levels in BALF between positive and negative groups of different pathogen types. **(A–F)** represents for the levels of IL-1Ra, IL-1Ra/IL-1α ratio, IL-18, IL-18/IL-38 ratio, IL-18BP and IL-18/IL-18BP ratio, respectively. **(G–L)** represents for the levels of IL-1α, IL-1α/IL-38 ratio, IL-1Ra/IL-1α ratio, IL-1Ra/ (IL-1α + IL-1β) ratio, IL-33 and IL-33/IL-38 ratio, respectively. The graph represents the median and interquartile range. * indicated *P* < 0.05, ** indicated *P* < 0.01. Virus + indicated virus positive, and virus- indicated virus negative. Bacteria + indicated bacteria positive, bacteria- indicated bacteria negative.

As shown in Figure 4, the IL-1Ra level, the IL-1Ra/IL-1*α* ratio, the IL-18/IL-38 ratio, the IL-18BP level, and the IL-18/IL-18BP ratio were significantly greater in the virus-positive group than in the virus-negative group (*P* < 0.05). Other cytokines and ratios were not significantly different (refer to Supplementary Figure S3). Compared with the bacteria-negative group, the bacteria-positive group presented significantly greater levels of IL-1*α* and a higher IL-1*α*/IL-38 ratio, whereas the IL-1Ra/IL-1*α* ratio, the IL-1Ra (IL-1*α* + IL-1β) ratio, the IL-33 level, and the IL-33/IL-38 ratio were significantly lower (*P* < 0.05). No other cytokines or ratios demonstrated significant differences (refer to Supplementary Figure S4).

#### 3.2.4 Comparison of cytokines in BALF between the Hi-positive and Hi-negative groups

**Figure 5:**
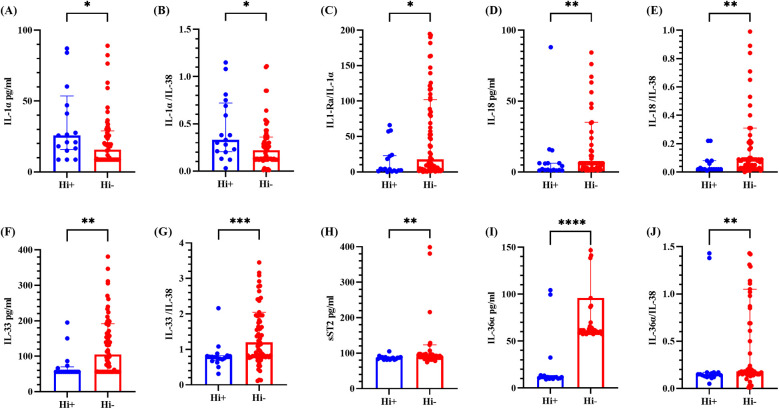
Cytokine levels in BALF from the Hi-positive and Hi-negative groups. **(A–E)** represents for the levels of IL-1α, IL-1α/IL-38 ratio, IL-1Ra/IL-1α ratio, IL-18 and IL-18/IL-38 ratio, respectively. **(F–J)** represents for the levels of IL-33, IL-33/IL-38 ratio, sST2, IL-36α and IL-36α/IL-38 ratio, respectively. The median and interquartile range are depicted. * denotes *P* < 0.05, ** denotes *P* < 0.01, *** denotes *P* < 0.001, **** denotes *P* < 0.0001, comparing the Hi-positive group and the Hi-negative group. Hi + indicated *Haemophilus influenzae* positive; Hi- indicated *Haemophilus influenzae* negative.

Owing to the highest detection rate of Hi within the bacterial group and its third-place ranking among all detected pathogens, it was separately classified for comparative analysis. As shown in Figure 5, the levels of IL-1*α* and the IL-1*α*/IL-38 ratio were elevated in the Hi-positive group compared with those in the Hi-negative group, which was consistent with the results from the bacteria-positive and bacteria-negative groups. Conversely, the IL-1Ra/IL-1*α* ratio, IL-18 level, IL-18/IL-38 ratio, sST2 level, IL-36*α* level and IL-36*α*/IL-38 ratio were diminished in the Hi-negative group. The IL-1Ra/IL-1*α* ratio, IL-33 level, and IL-33/IL-38 ratio findings were consistent with those observed in the bacteria-positive and bacteria-negative groups. All these differences were statistically significant (*P* < 0.05), whereas the differences in other cytokines and ratios were not statistically significant (refer to Supplementary Figure S5).

#### 3.2.5 Comparison of cytokines in the BALF of other groups of children

The IL-1β level in the corticosteroid-treated group [41.07 (11.94∼133.89) vs. 13.79 (5.97∼52.03) pg/ml] were significantly greater than those in the non-corticosteroid-treated group (*P* = 0.027 < 0.05). Similarly, the sST2 level in the immunoglobulin-treated group [91.63 (87.09∼634.53) vs. 87.09 (83.47∼96.16) pg/ml] were markedly elevated compared with those in the non-immunoglobulin-treated group, and the difference was statistically significant (*P* = 0.042 < 0.05). No statistically significant differences were observed in the other cytokines or ratios.

### 3.3 Predictive performance of the Il-18 level, the Il-18/Il-38 ratio, and the Il-33 level for diagnosing SCAP complicated with PE

**Figure 6:**
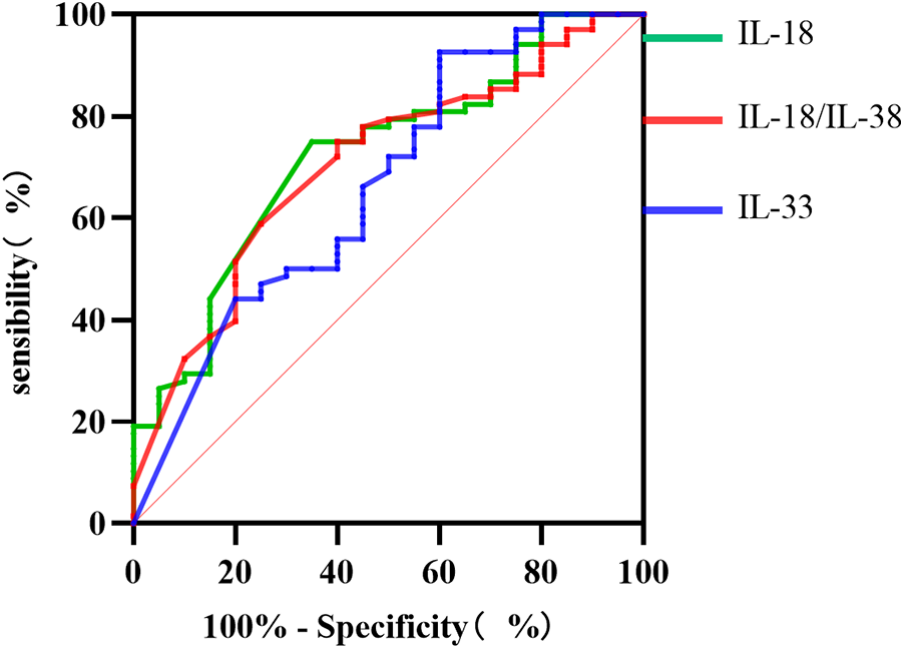
ROC curves for IL-18, the IL-18/IL-38 ratio, and IL-33.

As illustrated in Figure 6, the ROC curve results revealed that the IL-18 level, the IL-18/IL-38 ratio, and the IL-33 level exhibit substantial predictive efficacy for diagnosing SCAP combined with PE in children. The AUC values were 0.723, 0.705, and 0.664, respectively, with critical thresholds of 7.935 pg/ml, 0.130, and 217.755 pg/ml, respectively, each demonstrating high sensitivity and specificity.

## 4 Discussion

This study revealed elevated IL-18 level, IL-18/IL-38 ratio, and IL-33 level in the BALF of PE patients, with IL-18 demonstrating the greatest predictive efficacy. Another notable finding of this study is that significant differences in IL-36*α* level and the IL-36*α*/IL-38 ratio were found between the Hi-positive and Hi-negative groups, with the Hi-positive group showing lower levels (*P*-values <0.0001 and 0.0048).

According to statistics, approximately 20%–40% of hospitalized pneumonia patients will develop parapneumonic effusion (PPE) (15). PE is an independent risk factor associated with the failure of CAP treatment, and pneumonia patients with PE have a relatively high mortality rate (16). Therefore, identifying biomarkers for PPE prediction is crucial. IL-18 is a potent proinflammatory cytokine that modulates Th1 and Th2 immune responses, playing a critical role in inducing damage to various organs, including the lungs, liver, and intestines (17). It possesses high vascular permeability (18) and promotes fibrinolysis (19). A study by Rovina et al. (20) demonstrated that IL-18 is overexpressed locally in the pleural cavity of patients with empyema, which is correlated with increased pleural inflammation, whereas the serum IL-18 level was not significantly different. Rovina et al. (20) proposed that, given the attributes of IL-18, it is plausible that IL-18 could enhance the production of pleural effusion and foster pleural fibrosis associated with empyema. Notably, the BALF samples in this study also presented elevated IL-18 level, suggesting that IL-18 may be produced by resident pleural mesothelial cells or inflammatory cells or that there may be transmission of IL-18 or migration of related cells between the pleural cavity and the lungs. However, the potential sources and transmission of IL-18 remains somewhat speculative. Further investigation is needed.

IL-36 affects epithelial cells and specific immune cells, inducing cell activation and the secretion of cytokines and chemokines (21). IL-36 is expressed as an inactive precursor that requires proteolytic processing to become active. IL-36*α*, IL-36*β*, and IL-36*γ* are selectively activated by neutrophil granule-derived proteases, increasing their biological activity by approximately 500-fold (22). Research conducted by Ryffel et al. (23) demonstrated that IL-36*α* functions as a proinflammatory cytokine in the lungs, independent of IL-1*α* and IL-1β. Intratracheal administration of IL-36*α* induces neutrophil infiltration and enhances the expression of proinflammatory cytokines and chemokines in the lungs of both wild-type C57BL/6 mice and IL-1*α*- and IL-1β-deficient mice (23). Additionally, this study revealed significantly lower IL-18 levels, IL-18/IL-38 ratios, IL-33 levels, and IL-33/IL-38 ratios in the Hi-positive group than in the Hi-negative group.

*Haemophilus influenzae* (Hi), a pleomorphic gram-negative coccobacillus, is a unique human symbiotic pathogen capable of causing acute otitis media, sinusitis, pneumonia, and invasive diseases. In Portugal, the colonization rate of Hi in healthy children reaches 84.1% (24), whereas in China, a recent meta-analysis reported a carriage rate of 21% (25), with certain regions reporting rates as high as 59.91%. Among the 17 Hi-positive children in this study, 13 exhibited coinfections with other pathogens, implying that Hi may have been present prior to lung infection. Lindgren et al. (26) conducted a study on mice and revealed that those pretreated with non-typeable *Haemophilus influenzae* (NTHi) prior to infection with *Pseudomonas aeruginosa* presented diminished airway tissue damage and reduced inflammatory cytokine levels compared with those of those solely infected with *Pseudomonas aeruginosa*. NTHi markedly decreased susceptibility to subsequent *Pseudomonas aeruginosa* infection, likely due to the activation of the host's innate immunity rather than direct competitive interactions between species (26). While these findings indirectly support the findings of this study, further research is necessary to draw definitive conclusions.

To mitigate discrepancies in BALF sample recovery rates, this study endeavored to increase the ratio of proinflammatory cytokines to anti-inflammatory cytokines for intergroup comparisons. In this study, IL-38 was selected as the primary anti-inflammatory cytokine because it is a novel cytokine predominantly expressed in immune-related organs and is involved in various diseases, such as lung disorders, viral infections (27). Additionally, certain specific cytokine receptors function as inhibitors of proinflammatory cytokines (28). For example, IL-1Ra acts as an antagonist of IL-1*α* and IL-1β, IL-18BP serves as an antagonist of IL-18, and sST2 functions as an antagonist of IL-33. Consequently, in this study, some proinflammatory cytokines employed their respective antagonists as anti-inflammatory agents for ratio calculations. This study consequently introduced nine ratios (refer to section 2.5 Statistical analysis for details). The results revealed that, in most instances, intergroup differences in proinflammatory cytokines corresponded with variations in the ratio of proinflammatory to anti-inflammatory cytokines, suggesting minimal discrepancies in BALF recovery rates throughout the study.

Several limitations of this study warrant acknowledgment. First, the small sample size may introduce research bias, necessitating an increase in sample size to validate the clinical outcomes. Second, the concurrent infection of multiple pathogens and its impact on cytokine interactions were not considered; thus, further research is needed to confirm these experimental findings. In the future, we will collect additional samples, including plasma, pleural effusion, and BALF samples, to examine the predictive value of IL-18, the IL-18/IL-38 ratio, and IL-33 for PPE and to determine their potential as biomarkers for the differential diagnosis of PE and the rapid differentiation of pathogens, aiming to uncover more potential clinical applications. Concurrently, this study explored whether respiratory colonization by Hi can mitigate the production of inflammatory cytokines during pneumonia.

## 5 Conclusion

This preliminary study on children with SCAP suggested that detecting IL-18, IL-33, and IL-38 in the BALF may serve as valuable markers for predicting the occurrence of PE in these patients. Respiratory colonization by Hi may diminish the production of specific proinflammatory cytokines (IL-18, IL-33, and IL-36*α*) during the early stages of SCAP. Further sample collection, including plasma, pleural effusion, and BALF, is necessary to evaluate the predictive value of IL-18, the IL-18/IL-38 ratio, and IL-33 for PPE and to determine whether respiratory colonization by Hi can attenuate inflammatory cytokine production during pneumonia.

## Data Availability

The original contributions presented in the study are included in the article/Supplementary Material, further inquiries can be directed to the corresponding authors.

## References

[1] Jain S Williams DJ Arnold SR Ampofo K Bramley AM Reed C Community-Acquired pneumonia requiring hospitalization among U.S. Children. N Engl J Med. (2015) 372(9):835–45. 10.1056/NEJMoa1405870 25714161 PMC4697461

[2] Zar HJ Barnett W Stadler A Gardner-Lubbe S Myer L Nicol MP. Aetiology of childhood pneumonia in a well vaccinated South African birth cohort: a nested case-control study of the drakenstein child health study. Lancet Respir Med. (2016) 4(6):463–72. 10.1016/S2213-2600(16)00096-5 27117547 PMC4989125

[3] Pneumonia Etiology Research for Child Health Study G. Causes of severe pneumonia requiring hospital admission in children without HIV infection from Africa and Asia: the PERCH multi-country case-control study. Lancet. (2019) 394(10200):757–79. 10.1016/S0140-6736(19)30721-4 31257127 PMC6727070

[4] Yang M Meng F Gao M Cheng G Wang X. Cytokine signatures associate with disease severity in children with Mycoplasma pneumoniae pneumonia. Sci Rep. (2019) 9(1):17853. 10.1038/s41598-019-54313-9 31780733 PMC6882793

[5] Matarazzo L Hernandez Santana YE Walsh PT Fallon PG. The IL-1 cytokine family as custodians of barrier immunity. Cytokine. (2022) 154:155890. 10.1016/j.cyto.2022.155890 35462264

[6] Ge Y Huang M Yao YM. Recent advances in the biology of IL-1 family cytokines and their potential roles in development of sepsis. Cytokine Growth Factor Rev. (2019) 45:24–34. 10.1016/j.cytogfr.2018.12.004 30587411

[7] Dinarello CA. Overview of the IL-1 family in innate inflammation and acquired immunity. Immunol Rev. (2018) 281(1):8–27. 10.1111/imr.12621 29247995 PMC5756628

[8] Fields JK Gunther S Sundberg EJ. Structural basis of IL-1 family cytokine signaling. Front Immunol. (2019) 10:1412. 10.3389/fimmu.2019.01412 31281320 PMC6596353

[9] Martin SJ Frezza V Davidovich P Najda Z Clancy DM. IL-1 family cytokines serve as ‘activity recognition receptors’ for aberrant protease activity indicative of danger. Cytokine. (2022) 157:155935. 10.1016/j.cyto.2022.155935 35759924

[10] Declercq J De Leeuw E Lambrecht BN. Inflammasomes and IL-1 family cytokines in SARS-CoV-2 infection: from prognostic marker to therapeutic agent. Cytokine. (2022) 157:155934. 10.1016/j.cyto.2022.155934 35709568 PMC9170572

[11] Huang C Wang Y Li X Ren L Zhao J Hu Y Clinical features of patients infected with 2019 novel coronavirus in Wuhan, China. Lancet. (2020) 395(10223):497–506. 10.1016/S0140-6736(20)30183-5 31986264 PMC7159299

[12] Chen D Huang Y Jiao A Jin R Liu X Meng C. Expert consensus on the interventional diagnosis and treatment with respiratory endoscope in children with refractory pneumonia in China. Chin J Pract Pediatr. (2019) 34(06):449–57. 10.19538/j.ek2019060601

[13] Subspecialty Group of Respiratory Diseases SoP, Chinese Medical Association. Guidelines for the management of community-acquired pneumonia in children (2013 revision)(part 1). Chin J Pediatr. (2013) 51(10):745–52. 10.3760/cma.j.issn.0578-1310.2013.10.006 24406226

[14] Commission EgopredattotescoNH. Guideline of pediatric flexible bronchoscopy in China. Chin J Appl Clin Pediatr. (2018) 33(13):983–9. 10.3760/cma.j.issn.2095-428X.2018.13.006

[15] Light RW. Parapneumonic effusions and empyema. Proc Am Thorac Soc. (2006) 3(1):75–80. 10.1513/pats.200510-113JH 16493154

[16] Menendez R Torres A Zalacain R Aspa J Martin Villasclaras JJ Borderias L Risk factors of treatment failure in community acquired pneumonia: implications for disease outcome. Thorax. (2004) 59(11):960–5. 10.1136/thx.2003.017756 15516472 PMC1746855

[17] Vecchie A Bonaventura A Toldo S Dagna L Dinarello CA Abbate A. IL-18 and infections: is there a role for targeted therapies? J Cell Physiol. (2021) 236(3):1638–57. 10.1002/jcp.30008 32794180

[18] Vidal-Vanaclocha F Mendoza L Telleria N Salado C Valcarcel M Gallot N Clinical and experimental approaches to the pathophysiology of interleukin-18 in cancer progression. Cancer Metastasis Rev. (2006) 25(3):417–34. 10.1007/s10555-006-9013-3 17001512

[19] Meldrum KK Zhang H Hile KL Moldower LL Dong Z Meldrum DR. Profibrotic effect of interleukin-18 in HK-2 cells is dependent on stimulation of the toll-like receptor 4 (TLR4) promoter and increased TLR4 expression. J Biol Chem. (2012) 287(48):40391–9. 10.1074/jbc.M112.402420 23027874 PMC3504754

[20] Rovina N Dima E Psallidas I Moschos C Kollintza A Kalomenidis I. Interleukin-18 is up-regulated in infectious pleural effusions. Cytokine. (2013) 63(2):166–71. 10.1016/j.cyto.2013.04.017 23660216

[21] Gabay C Towne JE. Regulation and function of interleukin-36 cytokines in homeostasis and pathological conditions. J Leukocyte Biol. (2015) 97(4):645–52. 10.1189/jlb.3RI1014-495R 25673295

[22] Henry CM Sullivan GP Clancy DM Afonina IS Kulms D Martin SJ. Neutrophil-derived proteases escalate inflammation through activation of IL-36 family cytokines. Cell Rep. (2016) 14(4):708–22. 10.1016/j.celrep.2015.12.072 26776523

[23] Ryffel B Ramadas RA Ewart SL Iwakura Y Medoff BD LeVine AM. IL-36*α* Exerts pro-inflammatory effects in the lungs of mice. PLoS One. (2012) 7(9):e45784. 10.1371/journal.pone.0045784 23029241 PMC3447790

[24] Bajanca-Lavado MP Cavaco L Fernandes M Touret T Candeias C Simoes AS Haemophilus influenzae carriage among healthy children in Portugal, 2015–2019. Microorganisms. (2022) 10(10):1964. 10.3390/microorganisms10101964 36296240 PMC9611606

[25] Ma C Zhang Y Wang H. Characteristics of Haemophilus influenzae carriage among healthy children in China: a meta-analysis. Medicine (Baltimore). (2023) 102(44):e35313. 10.1097/MD.0000000000035313 37933036 PMC10627696

[26] Lindgren NR Novak L Hunt BC McDaniel MS Swords WE. Nontypeable Haemophilus influenzae infection impedes Pseudomonas aeruginosa colonization and persistence in mouse respiratory tract. Infect Immun. (2022) 90(2):e0056821. 10.1128/IAI.00568-21 34780275 PMC8853672

[27] Chen W Xi S Ke Y Lei Y. The emerging role of IL-38 in diseases: a comprehensive review. Immun Inflamm Dis. (2023) 11(8):e1287. 10.1002/iid3.1287 PMC10461426 37647430

[28] Shnayder NA Ashhotov AV Trefilova VV Nurgaliev ZA Novitsky MA Vaiman EE Cytokine imbalance as a biomarker of intervertebral disk degeneration. Int J Mol Sci. (2023) 24(3):2360. 10.3390/ijms24032360 36768679 PMC9917299

