# Supplementary material for: The role of IL-18 and IL-33 in the bronchoalveolar lavage fluid of children with severe community-acquired pneumonia complicated with pleural effusion
Source: Front Pediatr. 2025 Apr 25;13:1560328. doi: 10.3389/fped.2025.1560328 (PMC12063494; doi:10.3389/fped.2025.1560328)
Supplement: Supplementary file 1 [file Datasheet1.pdf]

Supplementary Material

1 Supplementary Figures

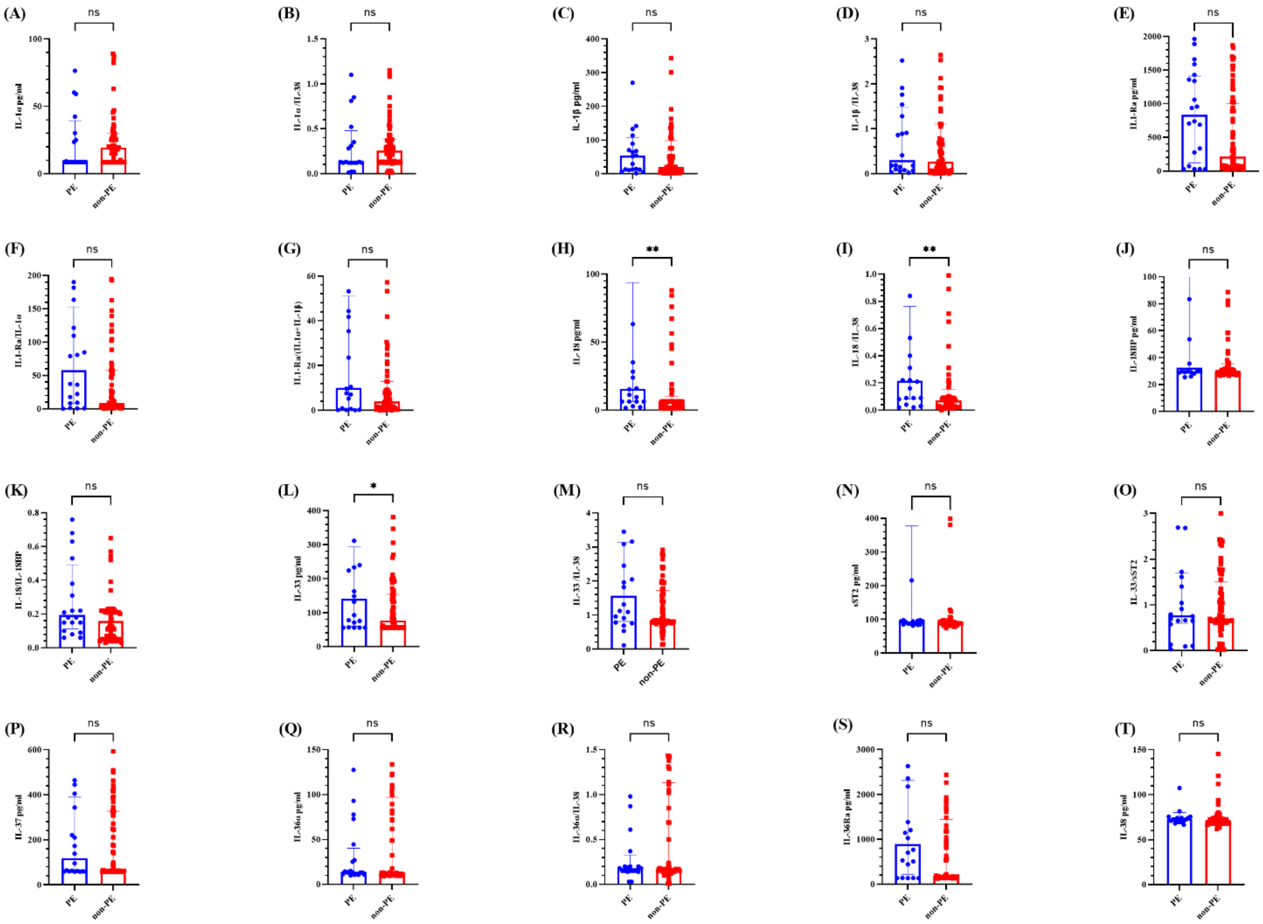

Figure S1. Cytokine levels in BALF from the PE and non-PE groups.

The figure displays the median and interquartile range. * denotes *P* <0.05, ** denotes *P* <0.01, comparing the PE and non-PE groups. PE stands for pleural effusion, and non-PE stands for no pleural effusion.

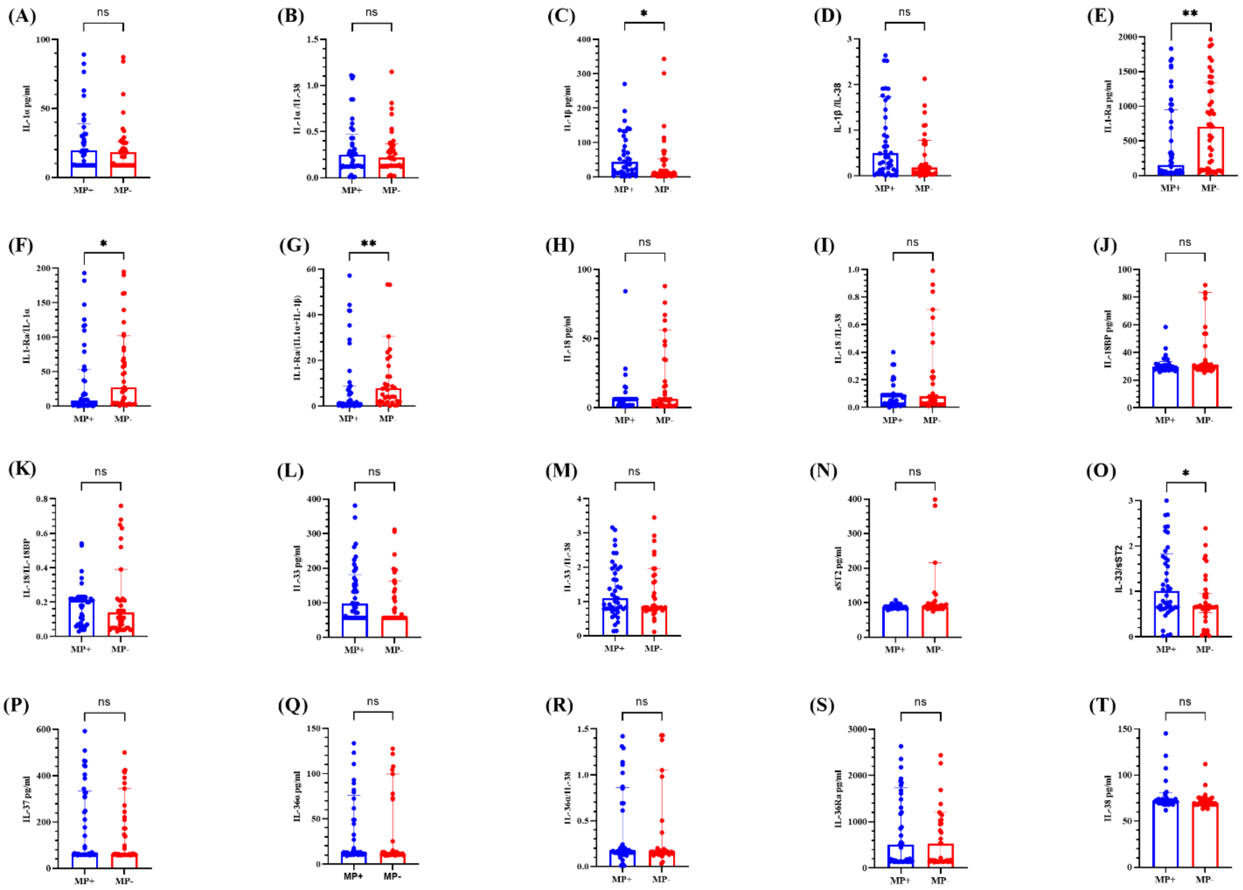

Figure S2. Cytokine levels in the BALF of the MP-positive and MP-negative groups.

The figure shows the median and interquartile range. An asterisk (*) denotes P <0.05, whereas a double asterisk (**) signifies P<0.01, comparing Mycoplasma pneumoniae-positive with Mycoplasma pneumoniae-negative groups. MP+ indicates *Mycoplasma pneumoniae*-positive; MP- indicates *Mycoplasma pneumoniae*-negative.

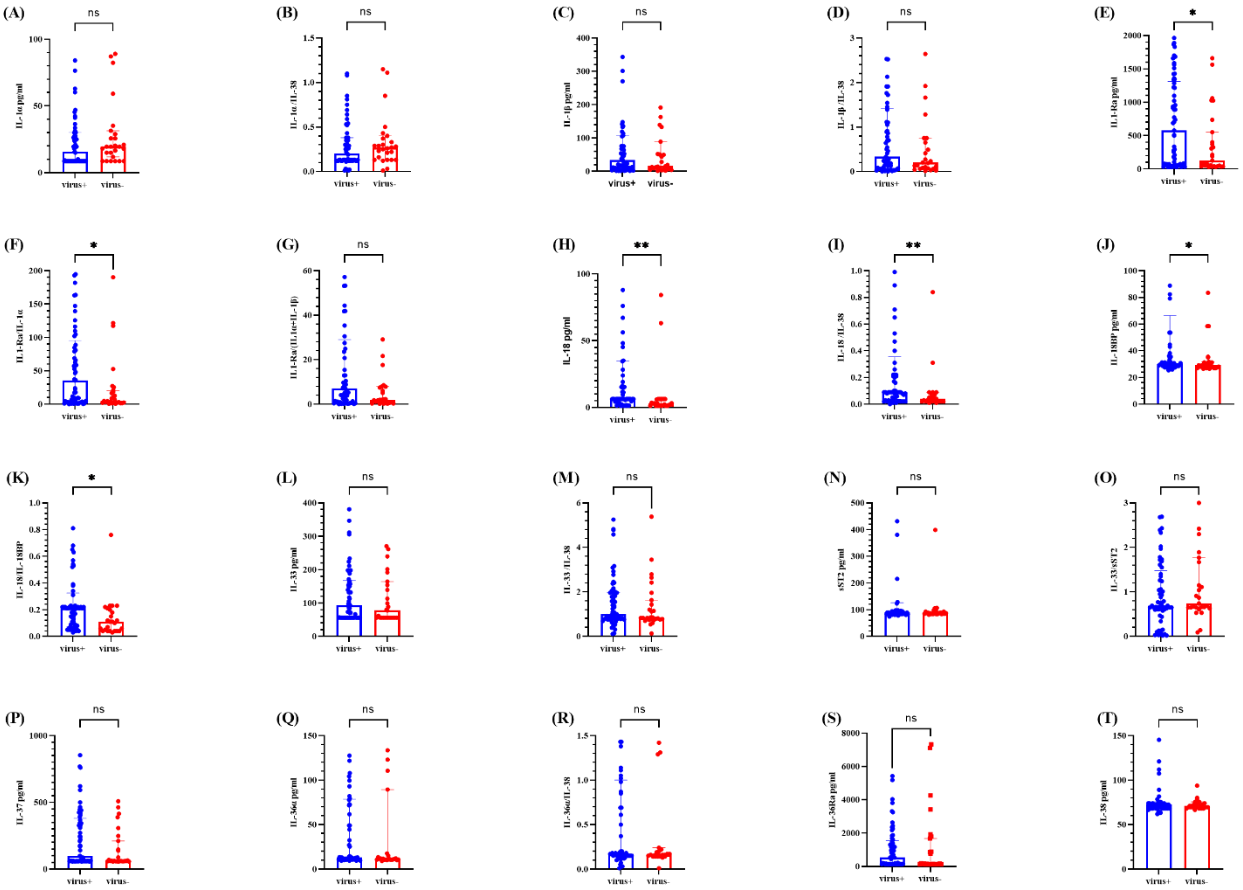

Figure S3. Cytokine levels in the BALF of the virus-positive and virus-negative groups.

The figure shows the median and interquartile range. * denotes P<0.05, ** denotes P <0.01, comparing the virus-positive group to the virus-negative group. Virus+ signifies virus-positive, and virus- signifies virus-negative.

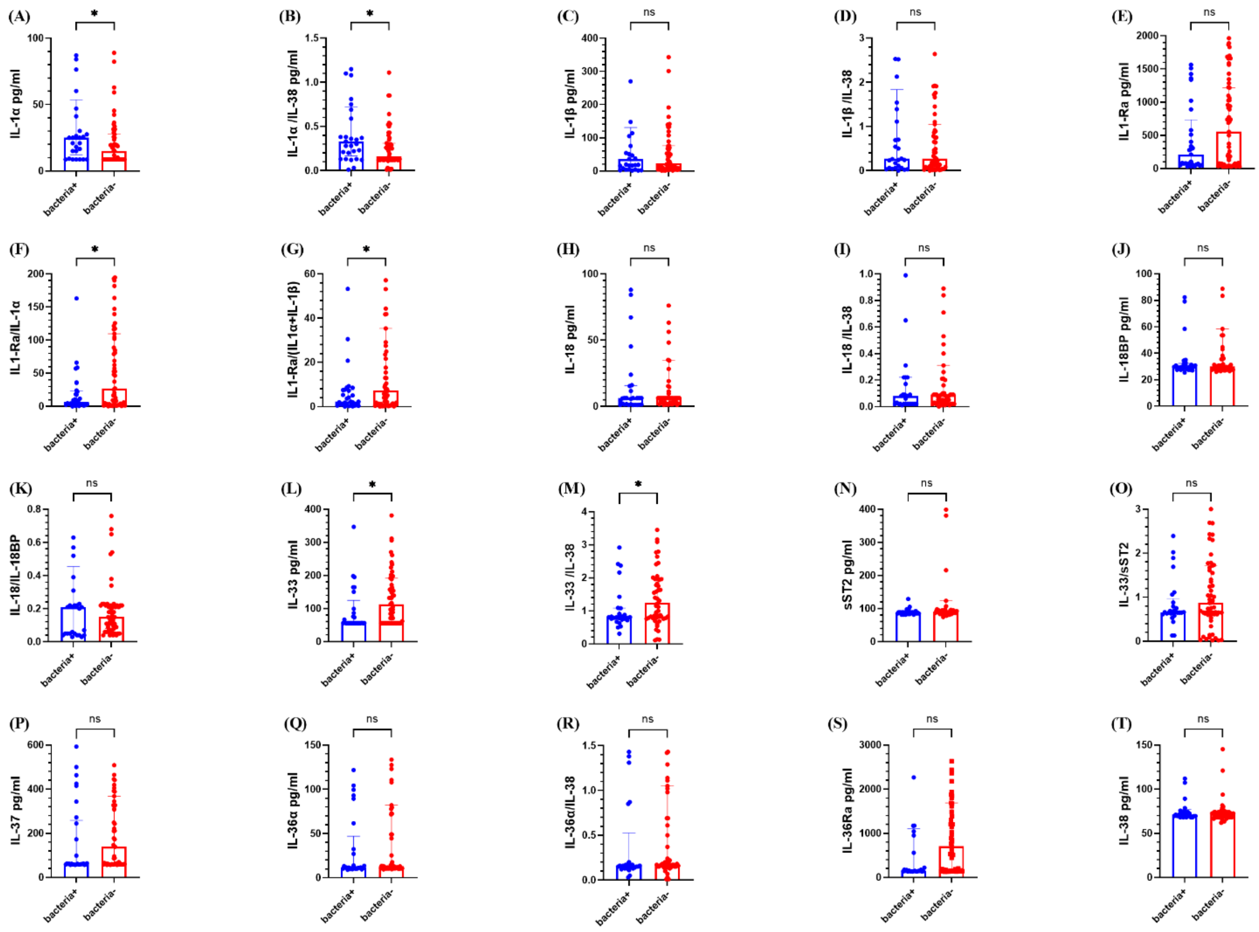

Figure S4. Cytokine levels in the BALF of bacteria-positive and bacteria-negative groups.

The figure displays the median and interquartile range. * denotes $P<0.05$, ** denotes $P<0.01$, comparing bacteria-positive and bacteria-negative groups. Bacteria+ indicates bacteria-positive bacteria, whereas bacteria- indicates bacteria-negative bacteria.

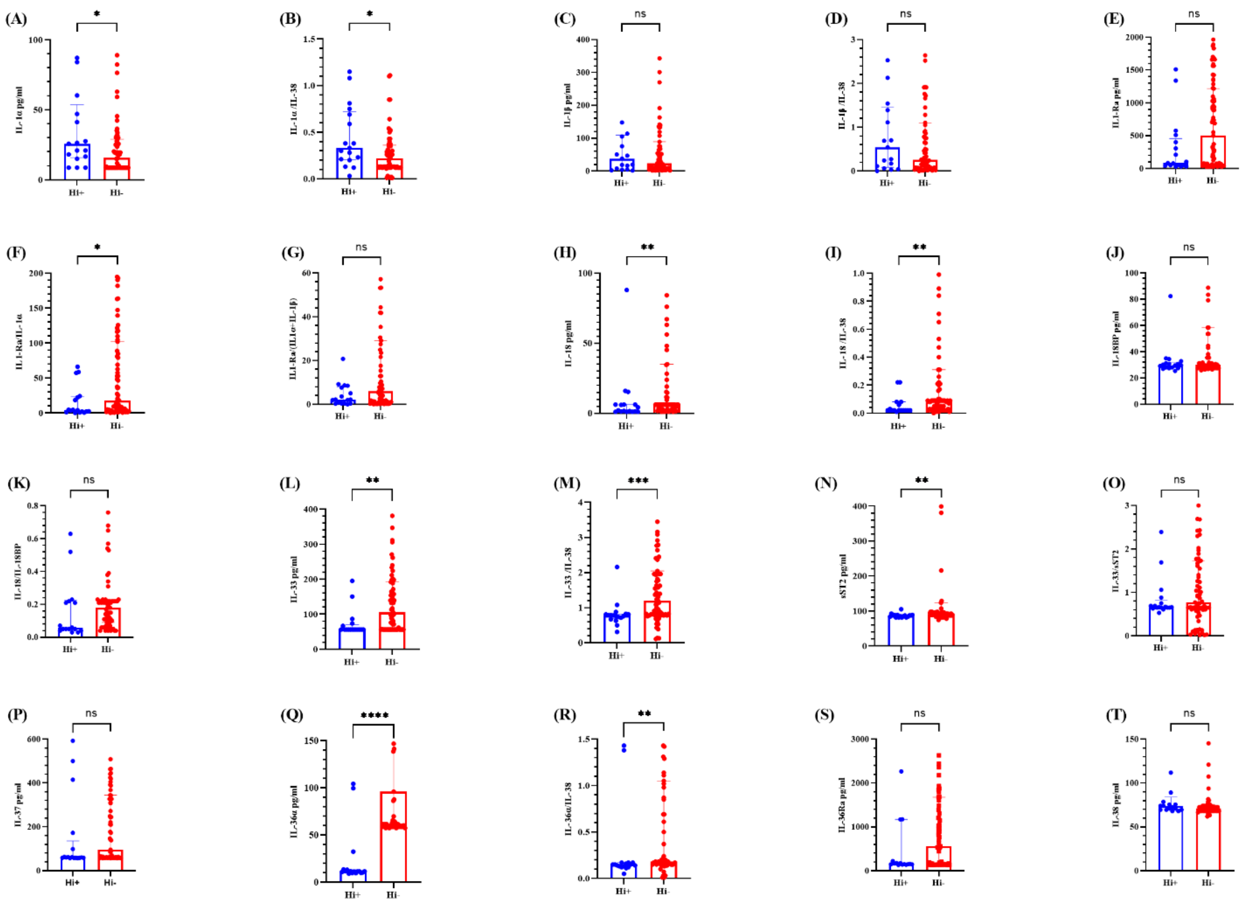

Figure S5. Cytokine levels in the BALF of the Hi-positive and Hi-negative groups.

The figure displays the median and interquartile range. * denotes $P<0.05$, ** denotes $P<0.01$, *** denotes $p<0.001$, and **** denotes $p<0.0001$, comparing the Hi-positive and Hi-negative groups. Hi+ indicates *Haemophilus influenzae* positive, whereas Hi- indicates *Haemophilus influenzae* negative.
